# Magnetic resonance imaging reveals functional anatomy and biomechanics of a living dragon tree

**DOI:** 10.1038/srep32685

**Published:** 2016-09-08

**Authors:** Linnea Hesse, Tom Masselter, Jochen Leupold, Nils Spengler, Thomas Speck, Jan Gerrit Korvink

**Affiliations:** 1 Plant Biomechanics Group and Botanic Garden, University of Freiburg, Germany; 2 Freiburg Centre for Interactive Materials and Bioinspired Technologies (FIT), Germany; 3 Competence Network Biomimetics, Germany; 4 Medical Physics, Department of Radiology, University Medical Center Freiburg, Germany; 5 Institute of Microstructure Technology, Karlsruhe Institute of Technology (KIT), Germany

## Abstract

Magnetic resonance imaging (MRI) was used to gain *in vivo* insight into load-induced displacements of inner plant tissues making a non-invasive and non-destructive stress and strain analysis possible. The central aim of this study was the identification of a possible load-adapted orientation of the vascular bundles and their fibre caps as the mechanically relevant tissue in branch-stem-attachments of *Dracaena marginata*. The complex three-dimensional deformations that occur during mechanical loading can be analysed on the basis of quasi-three-dimensional data representations of the outer surface, the inner tissue arrangement (meristem and vascular system), and the course of single vascular bundles within the branch-stem-attachment region. In addition, deformations of vascular bundles could be quantified manually and by using digital image correlation software. This combination of qualitative and quantitative stress and strain analysis leads to an improved understanding of the functional morphology and biomechanics of *D. marginata*, a plant that is used as a model organism for optimizing branched technical fibre-reinforced lightweight trusses in order to increase their load bearing capacity.

Technical fibre-reinforced structures find increased use in automobile, space and aircraft construction, windmills, sport devices, and recently also in architecture^1^,^2^,^3^,^4^,^5^. Their fibre-orientation can be targeted, which increases their load bearing capacity, decreases weight, and makes them less prone to failure^6^,^7^,^8^. However, delamination is still a problem which occurs especially in the nodal sections of branched fibre-reinforced structures, and can lead to a local failure, or even the collapse of the entire structure^9^,^10^ before the mechanical loading limits of the individual materials are reached. By optimizing the manufacturing processes and the technical fibre-reinforced component, these weak regions in branched elements can be reduced or even eliminated. A biomimetic approach using branchings of biological concept generators as a source of inspiration, allows for developing novel alternative design concepts, and technical structures that make full use of innovative insights gained through radical reconsideration of established design rules.

The branch-stem-attachments of the arborescent monocot genus *Dracaena* have been proven to be suitable concept generators for optimizing technical fibre-reinforced ramifications^6^,^7^,^11^,^12^,^13^,^14^,^15^,^16^,^17^ as their mechanically relevant tissue represents a fibre-matrix system that may serve as a role model for biomimetic branched lightweight components^11^,^16^. Therefore, it is crucial to understand how the branch-stem-attachments of *D. marginata* are adapted to mechanical loads and how the transfer of this knowledge into technical implementations can be performed most efficiently. The usual visualising methods, using histological techniques or micro–computed tomography (μCT), need extensive preparation and image-post-processing, and – most importantly – they are highly destructive (due to ionizing radiation, sectioning, or sample trimming) which prohibits stress-strain analyses^16^,^18^,^19^. Magnetic resonance imaging (MRI) can help to overcome these shortcomings, as this method allows to clearly differentiate various plant tissues without damaging the plant prior to or during image acquisition^20^,^21^,^22^,^23^. In this study, MRI will be used to gain insights into plant biomechanics and functional anatomy *in vivo* with the aim to qualitatively and quantitatively understand the complex three-dimensional deformations occurring in the plant body during mechanical loading. A special focus is laid on a possible load-adapted orientation of mechanical relevant tissues, and on understanding how stress peaks, occurring within the branch-stem-attachment region of *D. marginata*^13^,^14^, might be attenuated by a load-adapted orientation of vascular bundles with accompanying fibre caps.

## Results

### Image optimization

In our study, imaging needs to be performed with high spatial resolution in three spatial directions to image complex three-dimensional deformations of mechanical relevant tissues. Thus, 3D gradient echo sequences are the method of choice. Depending on the used parameters (flip angle α, repetition time TR, echo time TE) and optional RF-spoiling, images can either show T1-contrast (spin-lattice relaxation time) or a mixture of T1 and T2 contrast (spin-lattice and spin-spin relaxation times)^24^,^25^. Therefore, in order to allow for an efficient and ready segmentation of tissues in *Dracaena marginata,* T1 and T2 of the targeted tissues need to be determined. To accomplish this, detailed spin echo experiments for identifying intrinsic (T1 and T2) and operator-selectable parameters (e.g. resolution, TR and TE) were conducted^26^.

### Image contrast

Only spin echo images which were acquired using long repetition times (TR = 2000 ms) and long echo times (TE = 23.3 ms) created a noticeable contrast (T2-weighted) between vascular bundles and their fibre caps with respect to adjacent tissues in *Dracaena marginata* (Supplementary Fig. S1). The repetition time could be increased even further (TR ≥ 2000 ms) but this would also increase the image acquisition time with only a little gain in image quality. If the contrast is weighted towards the spin-lattice relaxation time T1 (TE = 7.7 ms; TR = 500 ms) or the proton density ρ (TE = 7.7 ms; TR = 2000 ms), the vascular bundles are not as clearly visible (Supplementary Fig. S1). T1-weighted images show almost no contrast between the vascular system and the adjacent parenchymatous ground tissues.

These findings were confirmed by the results of T1- and T2-relaxometry (Supplementary Fig. S2) using a spin echo sequence. Additionally, the parameter settings for TR and TE can be determined more precisely and help to further optimize the contrast of the images (TE = 39 ms and TR ≥ 1945 ms). The T1- and T2-relaxometry also shows that three tissues (the periderm, meristem and vascular bundles with fibre caps) show a similar spin-lattice relaxation time T1 (mean T1 relaxation times and standard deviations for these tissues are: *periderm*: 2224 ± 1.326 ms; *meristem*: 2391 ± 252 ms; *vascular bundles* 1941 ± 665 ms) and spin-spin relaxation time T2 (mean T2 relaxation times and standard deviations for these tissues are: *periderm*: 34.8 ± 3.1 ms; *meristem*: 37.5 ± 0.2 ms; *vascular bundles* 21.7 ± 1.3 ms). In order to clearly differentiate vascular bundles and the meristem, their respective signal intensity should differ significantly. This is only realized when using long repetition times (TR ≥ 1945 ms) and an echo time of 39 ms with both parameters leading to T2-weighted images (Supplementary Fig. S2).

### Image resolution

Three dominant parameters control the image resolution:

#### In-plane resolution

A high in-plane resolution (or small voxel size), is achieved by increasing the acquisition matrix (number of pixels in the frequency encoding and phase encoding direction), and keeping the field of view (FOV) as small as possible (i.e. the FOV only includes the region of interest). For all samples measured, an optimal in-plane resolution of 39 μm was determined (FOV = 1 cm × 1 cm, matrix = 256 × 256). Lowering the matrix size to 128 × 128 reduces the resolution to 78 μm. Increasing the FOV to 1.28 cm × 1.28 cm and lowering the matrix to 128 × 128 will decrease the in-plane resolution even further (Supplementary Fig. S3).

#### Slice thickness

Choosing a small slice thickness (voxel depth) will reduce the signal-to-noise ratio which deteriorates the quality of high-resolution images (slice thickness = 50 μm). If the slice is too thick, strong partial volume effects will occur and the image will become blurry (slice thickness = 600 μm). For *Dracaena marginata* a slice thickness between 100 μm and 200 μm leads to the best imaging results enabling an efficient and ready segmentation of the vascular bundles with fibre caps (phloem, xylem, sclerenchyma) (Supplementary Fig. S3).

#### Number of signal excitations (NEX)

Doubling the number of signal excitation (NEX; number of signals averaged) will increase the signal-to-noise ratio (SNR) by a factor of the square root of two. The disadvantage of large NEX is that it increases the image acquisition time by a factor of NEX. In practice, acquisition time is limited by system and sample stability and scanner availability. Therefore an intermediate number of excitations (NEX = 8; Supplementary Fig. S3) was chosen for all samples.

#### Transfer of image optimization results to biomechanical measurements

The results obtained by means of the spin echo experiments finally suggest performing T2 weighted imaging with high spatial resolution in three spatial directions. As this is not efficient with the vendor supplied spin echo sequences due to impractically long measurement times and either the need for much dead time in 3D sequences or low SNR in the 2D multislice sequences^27^, only 3D gradient echo offers a realistic approach. However, pure T2 weighting is not available here, which is why a non-RF-spoiled 3D gradient echo sequence was chosen as it offers mixed T1 and T2 contrast.

## *In vivo* visualisation of strains: Qualitative and quantitative deformation analysis

### Qualitative analysis of tissue displacements in plant ramifications

**Figure 1:**
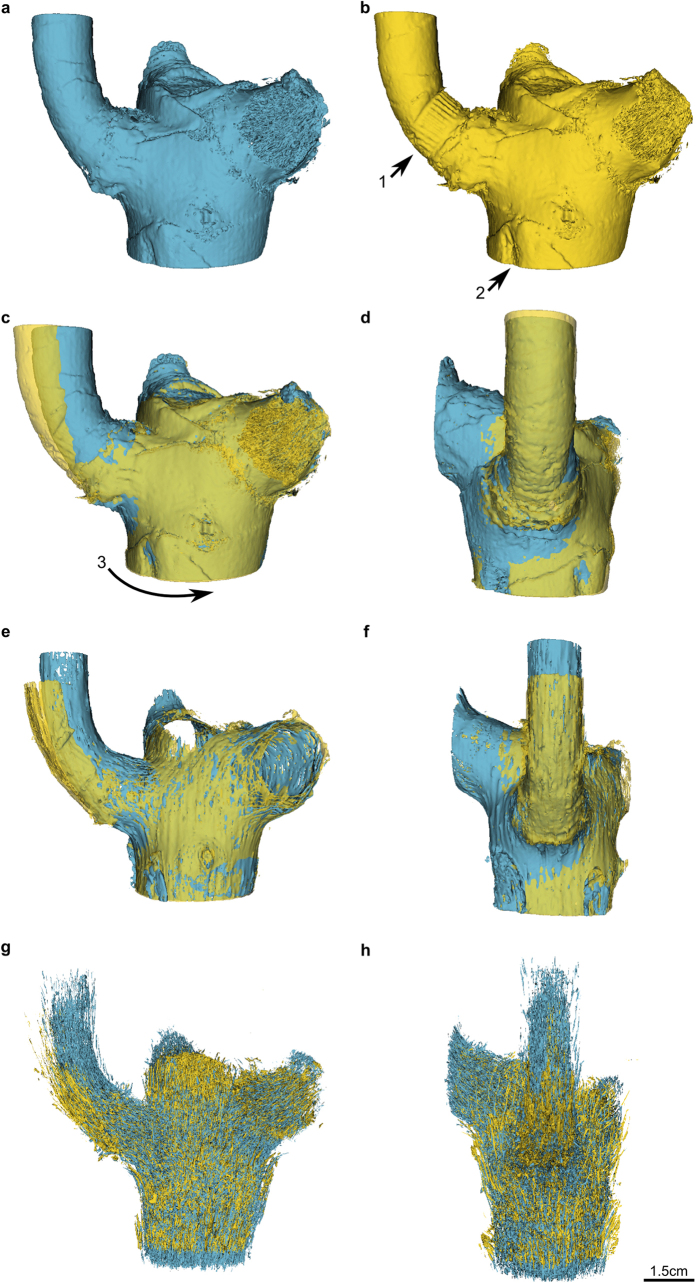
Quasi-3D data representations (3D models) of outer surface and inner tissues within the branch-stem-attachment of *Dracaena marginata* individual DM09. The models of the unloaded ramification are coloured blue; those of the loaded condition of the same ramification are coloured yellow. (**a**) Model of the outer surface of the unloaded ramification and (**b**) the same but mechanically loaded ramification. Injuries caused by the cable strap (arrow 1) and the plastic tip (arrow 2) of the experimental setup become visible. (**c**,**d**) A 3D model overlay of the outer surface of the ramification indicates a twisting of the entire plant as the branch bends outward (arrow 3). (**e**,**f**) The overlay of the meristem models show that the tissue deforms similarly to the outer surface. (**g**,**h**) Overlaying the models of the vascular system reveals that the detailed structure of the highly complex vascular system is very difficult to analyse.

A qualitative analysis of deformations is realised by creating quasi-3D data representations (further simplified to 3D models) of the outer surface, lateral meristem, vascular system and single vascular bundles of the branch-stem-attachment region of both individuals which are being compared in this study (DM09 and DM10). The deformation of the lateral meristem and the vascular system (the entity of every single vascular bundle and its fibre cap) are not being shown for individual DM10, as the content of information is low and is equally given with the 3D models of DM09 shown in Fig. 1.

**Figure 2:**
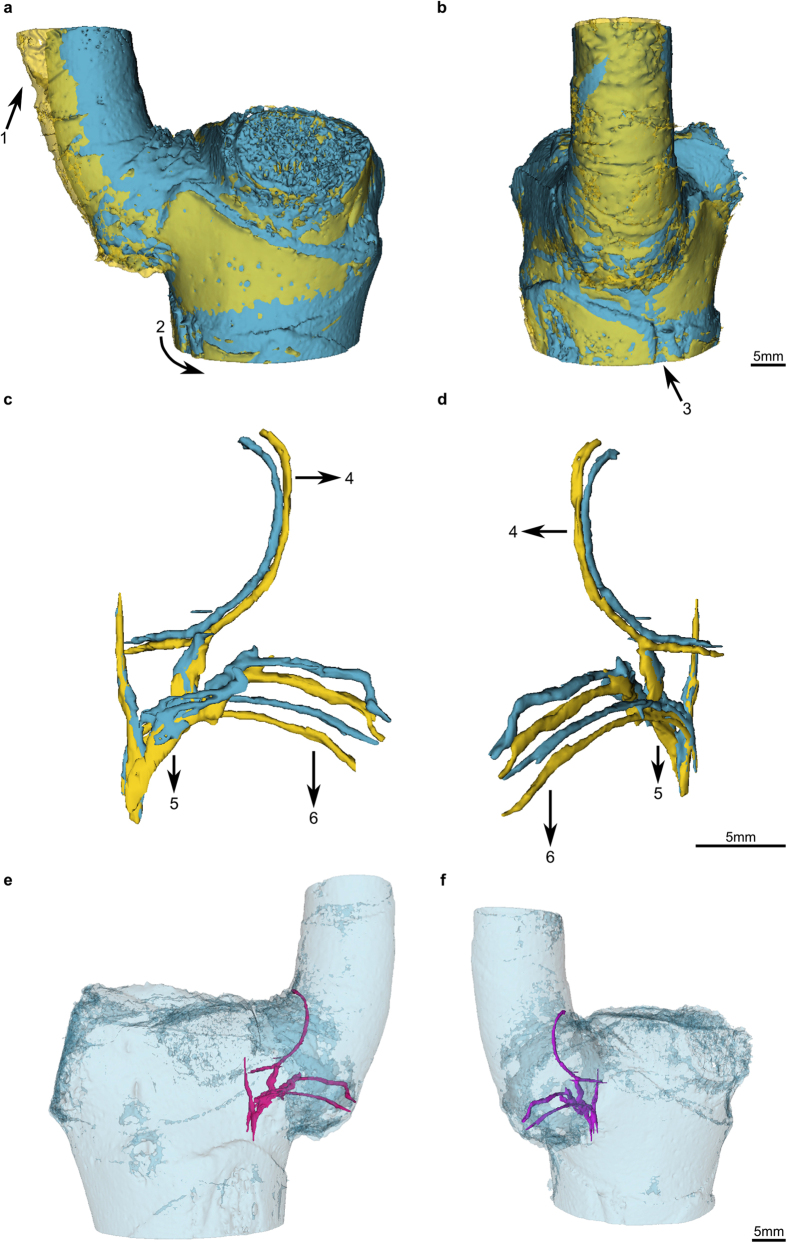
Quasi-3D data representation (3D models) of outer surface and single vascular bundles and their fibre caps within the branch-stem-attachment of *Dracaena marginata* individual DM10. The models of the unloaded ramification are coloured blue; those of the loaded condition of the same ramification are coloured yellow. (**a,b**) Overlay of the 3D models of the unloaded and loaded outer surface of the ramification. Injuries caused by the cable strap (arrow 1) and the plastic tip (arrow 3) of the experimental setup become visible. A slight twisting of the entire plant as the branch bends outward is indicated (arrow 2). (**c**,**d**) Models of single vascular bundles allow a detailed display and analysis of deformations. All vascular bundles are being bent downward. The displacements differ in their magnitude and are dependent on the location of the respective vascular bundle (arrows 4–6). (**e**,**f**) Spatial orientation of the unloaded vascular bundles.

The branch-stem-attachment region of both individuals does not only bend downwards, it is also being twisted counter clockwise during mechanical loading (Figs 1c–f and 2a,b). The displacement caused by twisting is greater for individual DM09. The reason for the torsion seems to be a combination of the asymmetric attachment of the branch to the main stem and the experimental setup. The deformations are especially apparent when comparing the unloaded and loaded outer surface of both individuals (Figs 1c,d and 2a,b) and the meristem of the ramification of DM09 (Fig. 1e,f). The deformations are less readily detected when comparing the unloaded and loaded vascular system of the ramification of DM09 (Fig. 1g,h).

**Figure 3:**
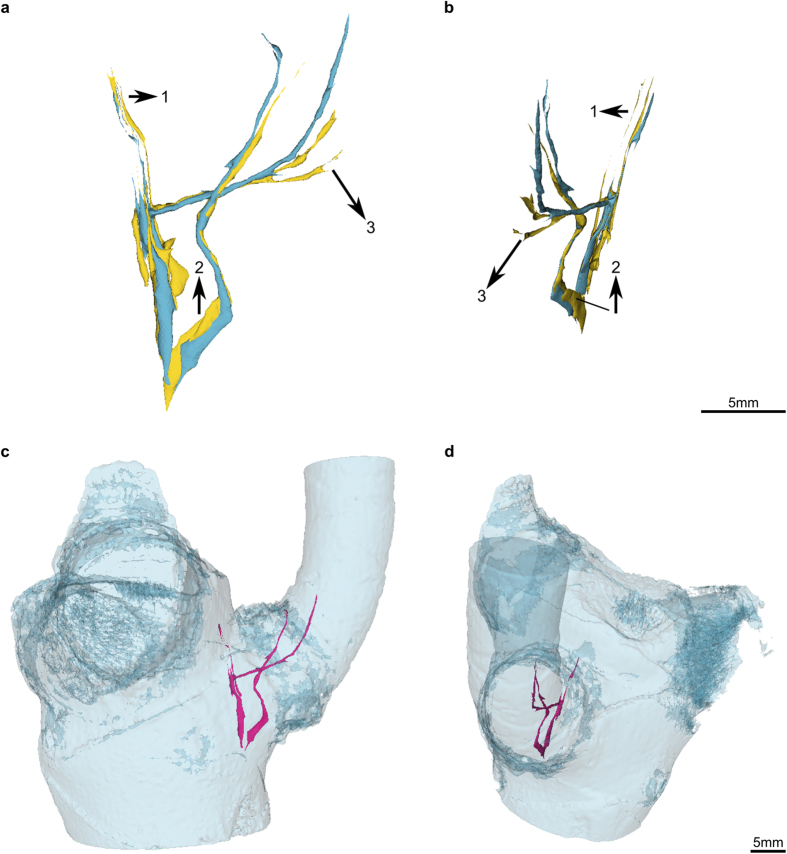
Quasi-3D data representation (3D models) of single vascular bundles and their orientation within the main stem of *Dracaena marginata* individual DM09. (**a**,**b**) The 3D models of single vascular bundles and their fibre caps allow a detailed display of deformations. The vascular bundles of the main stem (arrow 1) and the branch (arrow 3) bend outwards due to the applied force. An upward movement – contrary to the applied force – could be detected for the lower region of a vascular bundle in the branch-stem-attachment region (arrow 2). (**c**,**d**) Spatial orientation of the unloaded vascular bundles.

The deformations of single vascular bundles with fibre caps differ considerably between both individuals. The vascular bundles of the main stem and those of the branch of DM09 bend according to the applied load (arrows 1 and 3 in Fig. 3a,b). The displacement is greater in the branch than in the stem. Additionally, the torsion detected for the outer surface and the meristem also holds true for the vascular bundles. Furthermore, an upward movement contrary to the applied force could be detected for vascular bundle sections located closer to the main stem within the branch-stem-attachment region (Fig. 3, arrow 2 in a and b). The vascular bundles of DM10 also bend according to the applied load (arrows 4–6 in Fig. 2c,d). However, the displacement of the vascular bundles of the main stem is hardly existent whereas the displacements of the vascular bundles of the branch are explicitly greater and exclusively directed downward. Thus, the upward movement detected for vascular bundles of individual DM09 (Fig. 3, arrow 2 in a and b) are not present for the vascular bundles of individual DM10 (Fig. 2). The slight displacement detected for the outer surface caused by twisting is barely visible for the vascular bundles of individual DM10 shown in Fig. 2.

Artificial deformations caused by the experimental setup could be detected for both individuals. The cable strap incises the periderm, cortex tissue and lateral meristem and the constriction also affects the vascular system in this region. These deformations become visible when comparing 3D models of the plant outer surface in the unloaded and loaded situation (Figs 1 and 2). Furthermore, the plastic tip leads to indentations in the main stem (arrow 2 in Fig. 1b and arrow 3 in Fig. 2b). Thus, the experimental setup needs to be further optimized.

### Quantitative analysis of tissue displacements in plant ramifications

The manual determination of the displacement of characteristic features found along vascular bundles (turns, indentations, branchings, fusion, bumps etc.) was only sufficient for the y- (tension and compressive strain) and z-direction (shear or bending strain). This is due to difficulties of recognising the same features in the axial plane, which is crucial when determining displacements along the x-axis (shear and/or torsional strain).

#### Displacements along y-axis: Vy values

**Figure 4:**
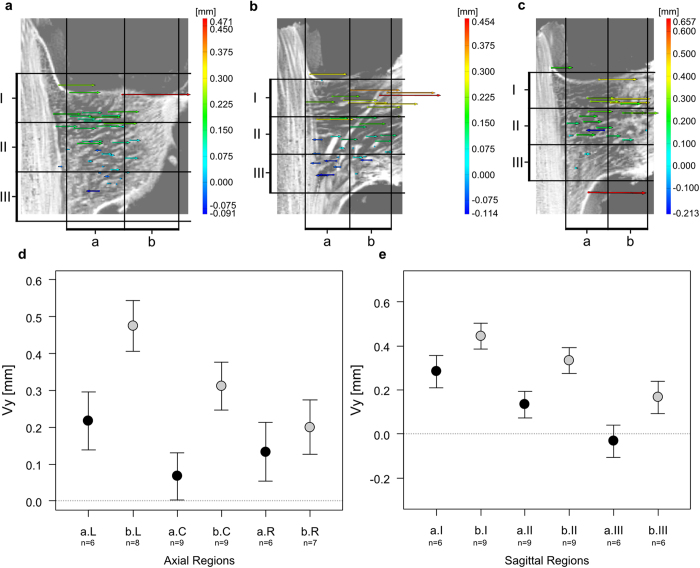
Software based and manual analysis of tensile or compressive strains of vascular bundles and their fibre caps for individual DM09. (**a**) Digital image correlation results of axial region left (L) of the branch-stem-attachment. (**b**) Digital image correlation results of axial region centre (C) of the branch-stem-attachment. (**c**) Digital image correlation results of axial region right (R) of the branch-stem-attachment. (**d**) Displacement along the y-axis (tensile and compressive strains) of characteristic features (branching, fusion, turns, bumps, indentations etc.) of vascular bundles and their fibre caps located in the coronal regions a or b in combination with their location in the axial regions left (L), centre (C) and right (R). Shown are the estimates and standard errors. (**e**) Displacement along the y-axis (tensile and compressive strains) of characteristic features of vascular bundles and their fibre caps located in the coronal regions a or b in combination with their location in the sagittal regions top (I), centre (II) and bottom (III). Shown are the estimates and standard errors. For an overview of the axial and sagittal regions also see Supplementary Fig. S4.

**Figure 5:**
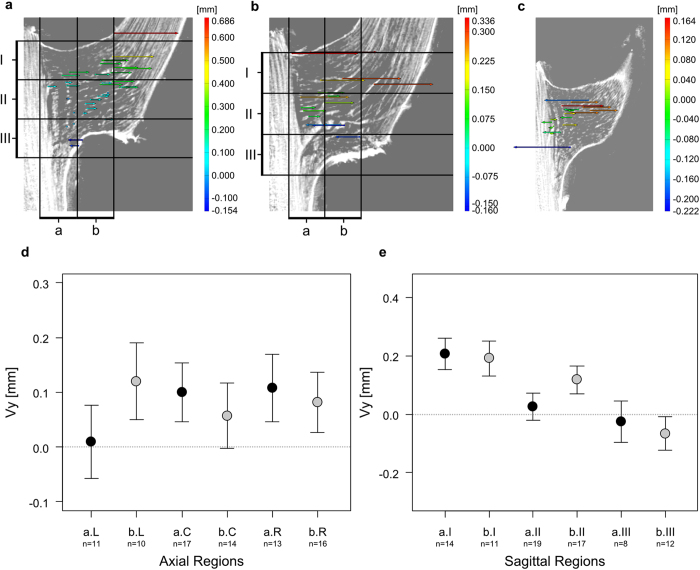
Software based and manual analysis of tensile or compressive strains of vascular bundles and their fibre caps for individual DM10. (**a**) Digital image correlation results of axial region left (L) of the branch-stem-attachment. (**b**) Digital image correlation results of axial region centre (C) of the branch-stem-attachment. (**c**) Digital image correlation results of axial region right (R) of the branch-stem-attachment. (**d**) Displacement along the y-axis (tensile and compressive strains) of characteristic features (branching, fusion, turns, bumps, indentations etc.) of vascular bundles and their fibre caps located in the coronal regions a or b in combination with their location in the axial regions left (L), centre (C) and right (R). Shown are the estimates and standard errors. (**e**) Displacement along the y-axis (tensile and compressive strains) of characteristic features of vascular bundles and their fibre caps located in the coronal regions a or b in combination with their location in the sagittal regions top (I), centre (II) and bottom (III). Shown are the estimates and standard errors. For an overview of the axial and sagittal regions also see Supplementary Fig. S4.

Deformations that occur along the y-axis give information’s concerning the outward pulling (tensile strains in direction away from the main stem; positive Vy values) or inward pushing (compressive strains towards the main stem; negative Vy values) of vascular bundles and their fibre caps. Within each individual these displacements can differ between axial-, coronal- or sagittal regions (Figs 4d,e and 5d,e, Supplementary Fig. S4).

Individual DM09: The displacements along the y-axis of vascular bundles within the branch-stem-attachment region of individual DM09 are almost exclusively directed outward (tensile strains; positive Vy values; Fig. 4d,e). Only some vascular bundles that are close to the main stem (coronal region a) and located in the centre and bottom of the branch (axial region C and sagittal region III) are being pushed inwards in direction towards the main stem (compressive strain; negative Vy values). This information is lost in Fig. 4d but becomes apparent in Fig. 4e. Vascular bundles located in the coronal region b are being displaced stronger into positive y direction (tensile strains) than vascular bundles located in coronal region a (b > a; ANOVA: Χ^2^(1) = 10.997, *p* = 0.001; *t* = 3.32, *p* = 0.002; Fig. 4d,e; Supplementary Table S1). The displacement of vascular bundles located in the coronal region b and axial region L (bL) is greater than those of the axial regions bC and bR (bC, *t* = −1.74, *p* = 0. 090; bR, *t* = −2.74, *p* = 0.009) indicating a gradual decrease of displacement within coronal region b from the left (L) to the right side of the branch (R). In addition, a gradual decreases of tensile displacements (0.05 < *p* < 0.1; Supplementary Table S1) could also be detected for both coronal regions from sagittal region I (top of the branch-stem-attachment) to the sagittal region III (bottom of the ramification). These differences are highly significant in coronal region a between aI and aIII (*t* = −3.06, *p* = 0.004) and in coronal region b between bI and bIII (t = −2.94, p = 0.006; Supplementary Table S1).

Individual DM10: The displacements along the y-axis (tensile or compressive strains) of vascular bundles within the branch-stem-attachment region of individual DM10 do not differ significantly between the axial- (L, C, R) and coronal regions (a and b; Fig. 5d; Χ^2^(5) = 1.94, *p* = 0.858). A displacement exclusively directed outward (tensile strains, positive Vy values) as found for individual DM09 cannot be proven for specimen DM10. However, the gradually decreasing displacement from the sagittal region I (top) to the sagittal region III (bottom) for all features located in coronal region a or b could also be shown for individual DM10 (Fig. 5e). These differences are significant for coronal region a (*p* < 0.05), but non-significant between vascular bundles of coronal region bI and bII (Supplementary Table S1).

#### Displacements along z-axis: Vz values

**Figure 6:**
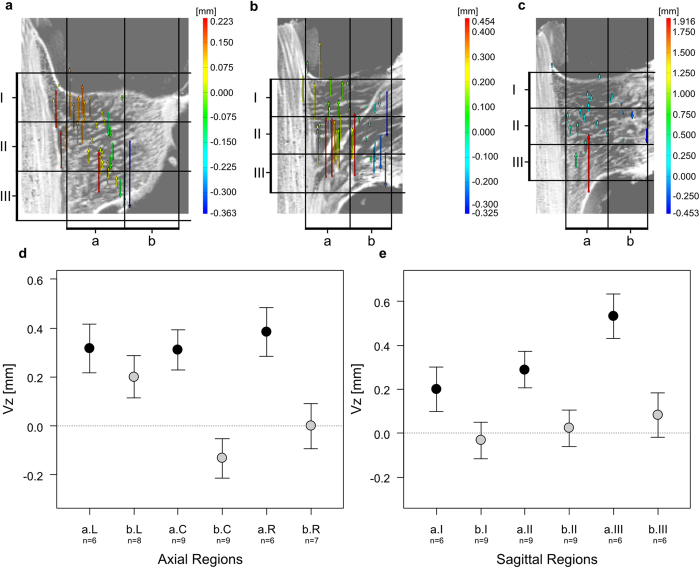
Software based and manual analysis of the displacement of vascular bundles and their fibre caps along the z-axis for individual DM09. (**a**) Digital image correlation results of axial region left (L) of the branch-stem-attachment. (**b**) Digital image correlation results of axial region centre (C) of the branch-stem-attachment. (**c**) Digital image correlation results of axial region right (R) of the branch-stem-attachment. (**d**) Displacement along the z-axis (shear and bending strains) of characteristic features of vascular bundles and their fibre caps located in the coronal regions a or b in combination with their location in the axial region left (L), centre (C) and right (R). Shown are the estimates and standard errors. (**e**) Displacement along the z-axis (shear and bending strains) of characteristic features of vascular bundles and their fibre caps located in the coronal regions a or b in combination with their location in the sagittal region top (I), centre (II) and bottom (III). Shown are the estimates and standard errors. For an overview of the axial and sagittal regions also see Supplementary Fig. S4.

**Figure 7:**
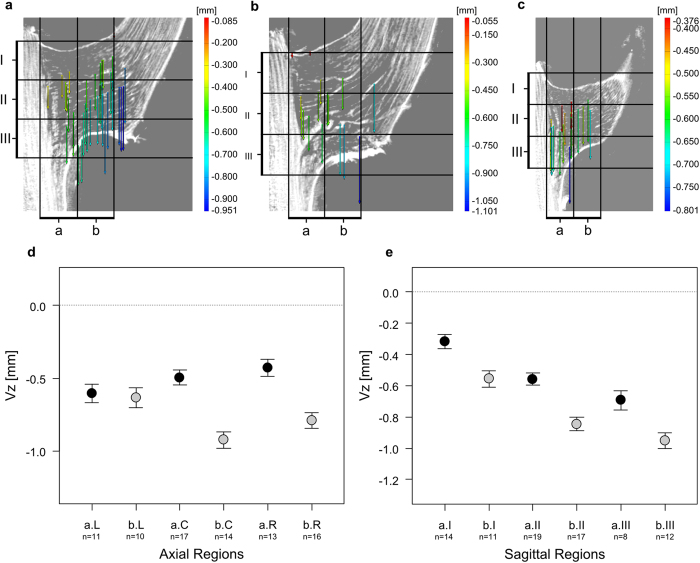
Software based and manual analysis of the displacement of vascular bundles and their fibre caps along the z-axis for individual DM10. (**a**) Digital image correlation results of axial region left (L) of the branch-stem-attachment. (**b**) Digital image correlation results of axial region centre (C) of the branch-stem-attachment. (**c**) Digital image correlation results of axial region right (R) of the branch-stem-attachment. (**d**) Displacement along the z-axis (shear and bending strains) of characteristic features of vascular bundles and their fibre caps located in the coronal regions a or b in combination with their location in the axial region left (L), centre (C) and right (R). Shown are the estimates and standard errors. (**e**) Displacement along the z-axis (shear and bending strains) of characteristic features of vascular bundles and their fibre caps located in the coronal regions a or b in combination with their location in the sagittal region top (I), centre (II) and bottom (III). Shown are the estimates and standard errors. For an overview of the axial and sagittal regions also see Supplementary Fig. S4.

Deformations that occur along the z-axis (shear and bending strains) give informations concerning the upward movement against the direction of applied loading (shear strain; positive Vz values) or downward movement in direction of applied loading (bending strain; negative Vz values) of vascular bundles and their fibre caps. Within each individual these displacements can differ between axial-, coronal- or sagittal regions (Figs 6d,e and 7d,e, Supplementary Fig. S4).

Individual DM09: The displacements along the z-axis of vascular bundles within the branch-stem-attachment region of individual DM09 differ highly significantly between coronal region a and b (Χ^2^(1) = 17.125, *p* < 0.001; Fig. 6d,e). Thus, vascular bundles and their fibre caps, which are located further away from the stem (coronal region b) are more likely being bent downward (negative Vz) than sheared upward (positive Vz; b < a; *t* = −4.14, p < 0.001). By contrast, vascular bundles, which are located closer to the main stem (coronal region a), are exclusively being sheared in an upward direction (positive Vz) against the direction of applied force (Fig. 6d,e). The downward displacement is strongest for vascular bundles of the central axial region, which are further away from the main stem (bC in Fig. 6d), and could also be detected for some vascular bundles of the axial region bR. Vascular bundles located in the regions bL tend to be rather sheared upwards than bent downwards when compared to those of the region bC (*t* = −2.81, *p* = 0.008; Supplamentary Table S1). The shear displacement (positive Vz values) gradually increases from the sagittal region I (top) to sagittal region III (bottom) (*t* = 2.32, *p* = 0.025 for aI and aIII). A corresponding increase for the coronal region b could not be proven statistically (Supplementary Table S1).

Individual DM10: The vascular bundles and their fibre caps of individual DM10 are exclusively being bent downward in direction of the applied load (negative Vz values; Fig. 7d,e). This downward displacement is significantly stronger (ANOVA: *Χ*^2^(1) = 34.32, *p* < 0.001) within the coronal region b (b < a; *t* = −5.86, p < 0.001;). Here, the displacement is strongest for vascular bundles of the axial region bC (Fig. 7d). The bending displacement of vascular bundles located in the axial region L and coronal region b seems to be smaller when compared to the regions bC and bR, which is comparable to the deformations of individual DM09. A gradual increase of a downward displacement (bending strain; negative Vz values) from the top (sagittal region I) to the bottom of the branch-stem-attachment (sagittal region III) could be statistically proven (Fig. 7e; Supplementary Table S1). This displacement is contrary to the findings concerning vascular bundles within the coronal region a of individual DM09 (Fig. 6e).

#### Quantitative analysis using digital image correlation

The findings of the manual deformation analysis match the results of the digital image correlation using the software ARAMIS Professional V8 SR1 (Figs 4, 5, 6 and 7a–c). Although some features that were targeted using the point based deformation analysis belong to the lateral meristem, all displacement vectors are within good approximation of the manual displacement measurements. However, each displacement vector needs to be considered separately and with care as the recognition of discrete features between both sagittal images of the different mechanical conditions of the branch-stem-attachment can include mistakes. Figure 4a additionally demonstrates the difficulties faced when determining characteristic features located in coronal region b, as the tissues are not as rich in contrast due to the constrictions caused by the experimental setup. Nevertheless, using both the results of the manual and software based deformation analysis gives sufficient quantitative insights into the displacement behaviour of the vascular bundles and their fibre caps.

#### Combined displacement: Deformations along y- and z-axis: Vy and Vz values

Visualising the combination of the displacement in y- (tensile and compressive strains; Vy values) and z-direction (shear and bending strains; Vz values) simultaneously, becomes possible using the digital image correlation software ARAMIS Professional V8 SR1 and by performing 2D elastic and consistent image registration using the Fiji plugin bUnwarp^28^,^29^. The vascular bundles of both individuals perform a clockwise rotation in branch direction around the bearing (where the branch is attached to the main stem). The results are exemplarily shown for the sagittal images of the axial region C for both individuals in Supplementary Fig. S6. The displacements of vascular bundles in the branch-stem-attachment of individual DM10 are comparable to those of DM09. However, the upward directed shear displacement is missing. Additionally, the rotation axis around the bearing is shifted further into the branch for individual DM09. Both automated software and manual analysis give comparable results.

## Discussion

In magnetic resonance imaging (MRI), the optimization of images is complex as imaging parameters can vary not only between species but also between individuals within one species due to differences in the individual age (young or old), growth condition (accessibility of light, water, nutrition and adequate temperature prior to image acquisition) and ecological background (abiotic and biotic stress during growth, i.e. intra- or interspecific competition, poor accessibility to resources etc.). There are far more parameters and sequences available to increase the image quality than those described in the current paper^27^. However, the aim of this method is not to visualise plant tissues on cellular level but to enable an efficient and ready segmentation of a tissue mix of the vascular bundles with fibre caps (phloem, xylem, sclerenchyma), the meristem (cambial tissue) and the periderm (parenchyma, phelloderm). With this respect, images can be evaluated simply by visual inspection. For visualizing the displacements and strains of the vascular bundles in the monocot *Dracaena marginata*, a high signal intensity of the vascular bundles is needed and the contrast to the surrounding tissues should also be high. The image contrast experiments show that a suitable contrast is achieved if the images are T2-weighted (Supplementary Figs S1 and S2). However, isotropic high spatial resolution T2-weighted imaging with spin echo sequences is not efficient, such that an efficient 3D gradient echo sequence showing mixed T1 and T2 contrast was used with parameters optimized according to the results obtained with the spin echo sequences. Performing image optimisation experiments is a necessary first step to a better understanding of how an adequate contrast can be created which clearly highlights the targeted tissues of interest. Furthermore, as MR-imaging cannot be considered being a standard procedure in plant biology, a consistent presentation of image optimization results can contribute to the development of a substantial data base future studies can refer to. With the presentation of our data we pursued the goal to also give a step-by-step approach enabling a simple, straightforward reproduction of the experiments with each kind of plant material. Additionally, using open access freeware for the image processing steps expands the usage of the method to a broader group of scientists.

**Figure 8:**
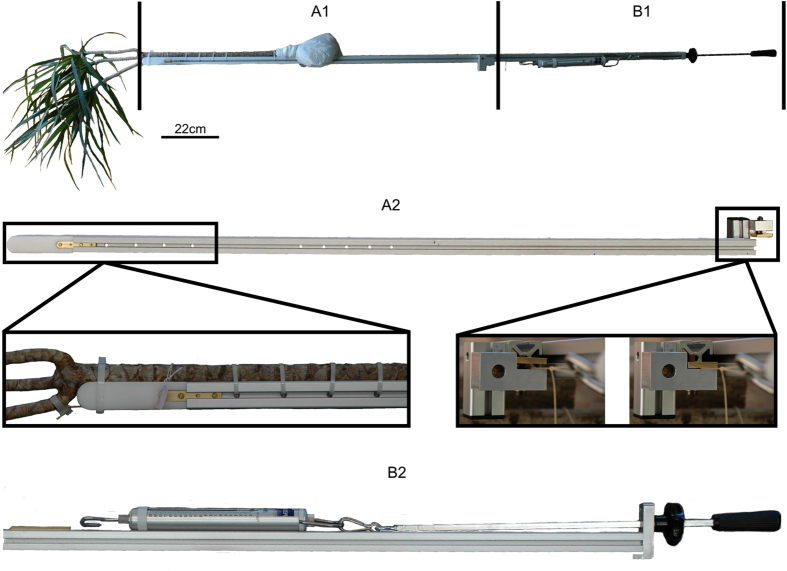
Experimental setup for applying mechanical loads to a ramification of *Dracaena marginata* during magnetic resonance imaging. The experimental setup is split into a non-magnetic skeletal structure (A1/A2) and a magnetic extension (B1/B2). A1/A2: A long aluminium beam is the base of the skeletal structure which also consists of a magnetic resonance imaging inactive plastic tip, two grooves for attaching the specimen and for guiding the load cable, holes for securing the specimen with cable straps and a bras-disk for locking the load cable into position after having applied a bending force. B1/B2: An aluminium beam, which can be attached to the skeletal structure, is the base of the extension. A holding for a spring scale, which is mounted to a screw handle, is fixed to the extension. The screw handle allows for precise adjustment of the applied force.

The experimental setup chosen for the biomechanical experiments integrated the spatial limitation of the MR-bore and other constraints imposed by the plants and the technical materials. During the biomechanical tests, special attention needs to be given to the attachment of the plant to the skeletal structure (Fig. 8, A1 and A2). Small deviations from the parallel alignment in particularly of the branch-stem-attachment to the aluminium beam can lead to larger twisting deformations of the entire ramification (Fig. 1c–f) which also include the vascular bundles and their fibre caps (Fig. 3a,b). Furthermore, the distance of the applied force to the branch in respect to the main stem has a significant effect on the occurrence of shear strains which superimpose the bending strains. This is not a new finding in the field of plant biomechanics where span-to-depth ratios were routinely calculated for tests of biological beams^30^,^31^,^32^. When testing the biomechanics of plant ramifications, however, comparable ratios need to be carefully considered. This is a crucial step as the influences of interior shear strains are barely apparent on the outer surface of the ramification (Fig. 1c,d) whereas they clearly affect single vascular bundles and their fibre caps (Fig. 3a,b). The differences of the vascular bundle displacements between individual DM09 and DM10 (compare Fig. 2c,d with Fig. 3a,b) can be led back to the varying lateral distance of the applied force to the main stem which is in the magnitude of roughly 1 cm (Supplementary Fig. S5). For experiments on mechanical loading of side branches in *Salix* and *Picea* both Beismann *et al*.^30^ and Müller *et al*.^33^ chose a distance of applied load from the main stem of approximately 1 cm, which is comparable to the distance chosen for individual DM09 showing the shear displacements (Figs 1, 3 and 6). However, the results of the electronic speckle pattern analysis of Müller *et al*.^33^ do not indicate shear displacements. Thus an appropriate distance of applied load should be determined individually for each species and for each individual specimen dependent on the morphometry of the branch-stem-attachment region.

The results of the biomechanical experiments reveal a load-adapted arrangement and orientation of the mechanical most relevant tissues, i.e. the vascular bundles and their fibre caps. In both individuals (DM09 and DM10) the occurring tensile strains (displacements into positive Vy values) are strongest in the upper region of the ramification (sagittal region I). In this region vascular bundles and their fibre caps are attached and orientated (nearly) perpendicular to the vascular bundles of the main stem and thus are placed parallel in direction of occurring stress and strains (Figs 2 and 3, Supplementary Fig. S5, and the refs 11, 13 and 14). In addition, in regions where compressive strains (negative Vy values) are highest (sagittal region III; Fig. 5e), vascular bundles describe a curve running up the main stem and progressively merge into the branch (Fig. 2, Supplementary Fig. S5, and the refs 11, 13 and 14). This curvature could promote a certain degree of mechanical flexibility within the branch-stem-attachment. On the one hand, compressive strains are “cushioned” as the vascular bundles are being pushed inward against the matrix of the sourrounding parenchyma tissue. On the other hand, the vascular bundles are being stretched simultaneously, reducing their curvature and aligning the mechanical relevant tissues parallel in direction with the bending or shear strains. If bending strains occur (Fig. 7) the vascular bundles located within the branch are being pulled in a downward direction, pressing the vascular bundles against the matrix of parenchyma tissue. Stresses and strains are being redirected along the length of the vascular bundles. If shear strains occur (Fig. 6) the vascular bundles located close to the main stem are being pulled upward in parallel to the main stem. Thus, stresses and strains are being redirected along vascular bundles of the main stem as vascular bundles of the branch-stem-attachment region have connectivity to vascular bundles of the main stem. The load-adapted placement and reorientation of vascular bundles and their fibre caps result in a high tensile resistance. In addition, the softer matrix of parenchyma tissue enables a certain degree of mechanical flexibility, as the tissue is compliant due to its pronounced visco-elastic behaviour. The geometry or outer surface of the ramification could have an additional load-adapted effect as the rotational axis of vascular bundle displacements around the bearing of the branch to the main stem is shifted further into the branch-stem-attachment region (see the results of individual DM09 in Supplementary Fig. S6).

The results of the biomechanical experiments could further allow a better and more detailed explanation of the failure modes observed by Masselter *et al*.^11^. While tensile stains (positive Vy values) in individual DM09 are slightly stronger within the upper sagittal region (I), the compressive strains (negative Vy values) increase from sagittal region I to sagittal region III (Fig. 4e). In combination with occurring shear strains (positive Vz values) in coronal region a (Fig. 6e) this could lead to the sickle-shaped detachment described by Masselter *et al*.^11^ once the applied load exceeds the structural strength of the ramification. This could further be promoted by the shift of the rotation axis of vascular bundle displacements into the branch caused by the geometry of the ramification (see the results of individual DM09 in Supplementary Fig. S6). Pure bending such as in individual DM10, however, could more likely lead to a fracture in the upper region (sagittal region I) of the branch (as described by Masselter *et al*.^11^) where tensile strains (positive Vy values) are stronger and combined with bending displacements (negative Vz values; Figs 5e and 7e). Thus, the upper region (sagittal region I) of the ramification could be considered as being the failure-prone region of the ramification as strong tensile strains are combined with bending or shear strains. This was also indicated by results of Haushahn *et al*.^13^.

Our method is a strong tool that can help for understanding the complex three-dimensional deformations of plant structures on different hierarchical levels, including the outer shape and the tissue arrangement in the ramification. A qualitative and quantitative observation of the strains of tissues and individual vascular bundles is possible and this *in situ* observation of the inner functional anatomy and its reaction to mechanical loading of a plant is thereby enabled for the first time (to the best knowledge of the authors). In addition, the abstraction of detailed (simulation) models of tissue arrangement and displacement becomes possible, which helps to understand the functional anatomy of plants in general and enables abstracting of interesting functional principles for a transfer in technical bio-inspired applications.

## Material and Methods

### Plant Material

Mature specimens of *Dracaena marginata* originated from commercial nurseries. All plants were kept in the greenhouse of the Botanic Garden of the University of Freiburg. Plants from commercial nurseries are typically decapitated to induce multifold branching (3–6 side branches develop at the tip of the main stem). The branches are located close to where the plant was decapitated (0–1 cm from the tip). The diameter of the main stem was between 1–4 cm and those of the branches varied accordingly between 0.5 and 1.5 cm. The roots were exempt from soil prior to investigation and wrapped with moist pulp (biomechanical testing) or entire smaller plants were transported along with their flower pots (image optimization). For MR-imaging, sufficient water content within the plant tissue is necessary, which is secured by adequate watering.

### Methods

#### Image optimization

Detected magnetic resonance signals depend on the natural and biological abundance of atoms having a nuclear magnetic moment (spin)^34^,^35^. In order to clearly image the branch-stem attachment of *Dracaena marginata in vivo*, magnetic resonance images in this study were generated from the hydrogen isotope 1H (proton). Magnetic resonance imaging is well established in medicine and a comprehensive database exists for finding suitable measurement parameters to match specific requirements. When imaging plant material, however, the adequate imaging sequence parameters first need to be determined. Detailed spin echo experiments for identifying intrinsic (T1 and T2) and operator-selectable parameters (e.g. resolution, TR and TE)^26^,^36^ were conducted using a Bruker Avance III Fourier Transform NMR spectrometer at the Karlsruhe Institute of Technology, which limits the sample size due to its small detection coil bore diameter (10 mm). Thus, four small stem segments of *Dracaena marginata* with a length between 1.5 cm and 2 cm and a diameter between 0.6 cm and 0.8 cm were measured. The aim was to determine parameters that enable the acquisition of images that clearly visualise and allow for an efficient and ready segmentation of the vascular system of *Dracaena marginata*.

#### Image contrast

A multi-slice multi echo (MSME) sequence was used to create an image series of the same slice with constant TR and varying TE (4.5–92.7 ms). MSME is based on a Carr Purcell Meiboom Gill Sequence (CPMG) where multiple spin echoes are generated. Each echo creates an image with a different TE. The experiment was repeated with varying TR (500–3000 ms; Supplementary Table S2).

The identification of the intrinsic parameters T1 and T2 for each tissue help to optimize TR and TE for enhancing the image contrast. In addition, the contrast of distinct tissues may be enhanced or suppressed, depending on their relaxation time, and T1 or T2-weighting enables the identification of regions and tissues with similar properties. Furthermore, the results of the MSME-experiments can be supported, which will help to understand how the contrast is generated. To determine T1 and T2 relaxation parameters, signal intensities were recorded at six different TRs (245 ms, 445 ms, 745 ms, 1145 ms, 1545 ms, and 1945 ms) and six different TEs (10 ms, 20 ms, 30 ms, 40 ms, and 50 ms). Observing a field of view (FOV) of 1 cm × 1 cm and applying a matrix (MTX) of 256 × 256 resulted in an in-plane resolution of 39 μm × 39 μm, while a slice thickness of 0.3 mm was used. Due to the small feature sizes of the vascular bundles and of the periderm, signal intensities were extracted from only one and four (two by two) voxels, respectively, while a region of interest (ROI) of nine (three by three) voxels was set for the remaining three tissues (cortex, meristem, parenchyma). Due to the possibility of the matrix only partially covering a vascular bundle, seven different vascular bundles were investigated, whereas for the remaining tissues two different regions were considered per tissue. Finally, the different values were averaged, before an exponential fitting function was applied in the form of y = A + C*exp(−t/T2) for T2 decay and y = A + C*(1 − exp(−t/T1)) for T1 recovery. The data were fitted by the vendor (Bruker) supplied “image analysis tool” using a Levenberg-Marquardt non-linear least squares algorithm.

#### Image resolution

The image quality also strongly depends on the image resolution. Three operator selectable parameters have a dominant effect on the image resolution.

In-plane resolution: A spin echo sequence (MSME, with increasing TE) was applied to determine an ideal in-plane resolution with only little decrease of the signal intensity. Three measurements were conducted with varying field of view (FOV) (spatial encoding or square image area containing the region of interest measured) and acquisition matrix (number of pixels in the frequency encoding and phase encoding direction). All of the other operator selectable parameters were held constant (Supplementary Table S2).

Slice thickness: The size of a volume element (voxel) scales with the slice thickness (voxel depth) and by this with the strength of a signal detected from this voxel. An ideal slice thickness was determined by repeatedly acquiring images using MSME with increasing TE and varying slice thickness (50 μm–600 μm) but keeping all of the other operator selectable parameters constant (Supplementary Table S2).

Number of signal excitation (NEX): The quality of an image can be increased further by reducing the signal-to-noise ratio (background disturbance lowering the image quality). This is achieved by averaging a number of measured signals (the number of signal excitation, NEX). An ideal NEX was determined by repeatedly acquiring images with a spin echo sequence (MSME) with increasing TE and varying NEX (4–16), but keeping all of the other operator-selectable parameters constant (Supplementary Table S2).

### Deformation analysis of plant ramifications

The results of the parameter determination are used for *in vivo* imaging while mechanically testing the branch-stem junctions of *Dracaena marginata*.

#### Experimental setup

To examine how mechanical loading strains the vascular system as the mechanically most relevant tissue, a branch-stem-attachment needs to be imaged under unloaded and subsequently under a loaded condition. For this, an experimental setup had to be designed which applies static mechanical loading to the ramification for a time duration of 9–15 h and can be used within and/or close to strong magnetic fields.

A square, non-magnetic aluminium alloy beam with a length of 119 cm and a width of 3 cm provides the main support of the setup (Fig. 8). Two notches were milled into the aluminium of which one is for fastening the main stem of a *Dracaena marginata* sample and the other notch is to guide the load cable (Kevlar aramid fibre rope, Jutta Lingen SÜDTEX-Vertrieb Skytex, Germany). A non-magnetic plastic extension (length: 11.5 cm; width: 3 cm) at the tip of the aluminium beam located close to the region of interest (ROI) prevents magnetic inhomogeneity artefacts caused by magnetic properties of the aluminium alloy beam. The branch of the sample is located at the tip of the plastic extension and faces the notch for the load cable guidance. The load cable is attached to a cable strap, which is fixed to the branch on one side and a spring scale (20 kg spring scale, PESOLA Macro Line - Swiss made, Switzerland) on the opposite side (Fig. 8). A brass-disc for locking the load cable into position is attached to the aluminium beam via brass screws and located at the opposite side of the plastic tip (Fig. 8, A2). All components attached to the skeletal structure are non-magnetic. An extension consisting of a second aluminium beam, attachments for the spring scale and brass-fixations to attach the extension to the skeletal structure holds magnetic components (Fig. 8, B1 and B2). This extension serves to apply mechanical loads to the ramification and can be demounted after the load cable is locked by the brass-disk of the skeletal structure.

#### *In vivo* biomechanical experiments

In this study two individuals of *Dracaena marginata* (DM09 and DM10) are being compared. Both individuals differ due to the location of the applied load. Individual DM09 was loaded close to the main stem (distance main stem to load cord: abaxial = 8.5 mm; adaxial = 10.5 mm), whereas the mechanical loading of individual DM10 was shifted along the branch further away from the main stem (distance main stem to load cord: abaxial = 34.1 mm; adaxial = 24.7 mm; see Supplementary Fig. S4).

Before image acquisition the entire *Dracaena marginata* plant is attached to the skeletal structure via non-magnetic cable straps (Fig. 8, A1 and A2). The roots are held moist by wrapping them into initially wet pulp and a plastic bag, which is closely strapped around the base of the main stem. All branches but the biomechanically tested ramifications are cut down leaving 2–3 cm stubs. This helps to locate the mechanically loaded branch-stem junction after image acquisition and increases the space within the radio frequency coil, which is necessary for mechanical loading. The plant and the skeletal structure are placed into the bore of the MR-unit and the ramification is imaged under unloaded conditions.

Subsequently, the plant is retrieved out of the MR-unit and the extension (Fig. 8, B1 and B2) is attached to the skeletal structure. The ramification is mechanically loaded by attaching the load cord to the side branch and the spring scale and pulling the extension axially away from the ramification until a load of 150 N acts on a cable strap that connects the load cord to the side branch. Due to the mechanical load, the cable strap incises into the cortex, which leads to tissue compression and injuries in this region of the branch. Possible creeping effects and the injuries lead to a decrease of the applied force within the first hour of mechanical loading. This is why the ramification is mechanically loaded prior to image acquisition and the applied force is readjusted. In a next step, the load cord is locked into position by the brass-disc and the extension is demounted. The aluminium alloy beam and the mechanically loaded ramification are placed into the MR-unit and an image of the mechanically loaded state of the ramification is acquired. After these biomechanical experiments the specimen is placed back into soil and can continue its growth.

MR- imaging was performed with a 9.4 T Bruker Biospec 94/20 small animal scanner located at the University Medical Center Freiburg equipped with a quadrature volume coil with 7 cm inner diameter. This scanner was chosen because the 11.7 T scanner is not suitable to contain the whole plant, due to insufficient bore diameter. Images were acquired using a 3D FLASH sequence (gradient echo) that was optimized based on the T1 and T2 values obtained via the spin echo relaxometry on the 11.7 T scanner and led to the following parameters: TR = 26 ms, TE = 6.5 ms, matrix size 512 × 67 × 448 voxels, voxel size 98 × 98 × 100 μm^3^, NEX = 6, RF-spoiling off, acquisition time 13 h 7 min. The flip angle was 50° to obtain contrast maximum of vascular bundles and peridem with 11.7 T T1/T2 ratios of 1783/24 and 1286/37, respectively. The specific gradient echo sequence was chosen for high-resolution 3D imaging, the long acquisition time provides a suitable signal-to-noise ratio (SNR)^24^.

#### Image-post processing for a qualitative deformation analysis

The image raw-data is exported as DICOM image series (.dcm). To fully understand the influence of mechanical loading on plant ramifications, its geometry and tissues (meristem and mechanically relevant vascular bundles with fibre caps) need to be visualised in a three-dimensional way without disturbing adjacent tissues. This called for the use of several segmentation and analysis tools that are described below.

ITK-SNAP: image segmentation: ITK-SNAP^37^ (Version 3.0.0) is an open-source software for creating segmentation images from medical image files (e.g. DICOM image series, or NiFTI files). The semi-automatic segmentation tool (active contour segmentation (snake) mode) allows the operator to control which structures should be segmented. The segmentation image can be altered or enhanced by manual segmentation (paintbrush mode).

ITK-SNAP was used to generate 3D segmentations of the vascular system and the surrounding meristematic tissue (the single steps are described in more detail in the Supplementary method section online). The imported raw-data (DICOM format; dcm) can be exported as main images in NiFTI format (.nii). The segmentation images are exported separately as NiFTI-files (.nii).

3D-Slicer: image analysis: It is crucial to reproduce the exact location and orientation of the ramification in the loaded condition. Therefore, the resulting images of both mechanical situations (unloaded and loaded) had to be aligned and reoriented to create an image overlay. For this, the main images (raw-data in NiFTI format; nii) and the image segmentation results (segmentation images in nii format) are exported from ITK-SNAP and imported into 3D-Slicer (Version 4.3.1). 3D-Slicer is an open source software package for image analysis and visualization^38^. The main image (.nii) of the unloaded ramification was used as a reference image. All images of the ramification under mechanical load (main image and segmentation images) were aligned and reoriented according to the reference using the transforms module of 3D-Slicer. Subsequently, in a step-by-step approach (see Supplementary method section online), possible changes of the outer surface, the vascular system, the meristematic tissue and specific individual vascular bundles were examined.

Geometrical changes of the ramification during mechanical loading: The main images of the ramification in an unloaded and loaded condition were converted into 3D-models of the plant ramification in order to visualise the geometrical changes of the branch-stem-attachment during mechanical loading. For this purpose, a fast and automated segmentation of the entire plant structure was realized using the threshold effect of the editor module by altering the threshold modulator. The threshold modulator sets the lower and upper threshold boundaries. The lower threshold boundary is altered until no background noise is included into the label map and only the geometry of the ramification is displayed. The models were constructed with the model-maker module using the label map volumes created with the editor module. The models can then be displayed in the 3D-viewer of 3D-Slicer and the 3D deformations become visible.

Changes of the meristem and the vascular system during mechanical loading: The segmentation images of the meristem and the vascular system were converted into 3D-models according to the procedure of the models of the outer shape.

Changes of particular vascular bundles during mechanical loading: Specific vascular bundles with their fibre caps in both situations (loaded and unloaded) were manually segmented in 3D-Slicer using the paint effect of the editor module. For the identification of identical vascular bundles, 3D-Slicer is especially suited due to the possibility of an exact overlay of the image raw-data. The segmentation images of the vascular bundles were converted into models according to the procedure of the earlier models.

An ideal identification of 3D deformations is achieved when creating overlays of the models to directly compare the unloaded and loaded situations. The visualization of the models and their opacity can be altered using the model module in 3D-Slicer. These unique 3D images help understanding the effects of mechanical loading in a plant ramification on three hierarchical levels: the overall outer shape of the ramification, the structure and arrangement of inner tissues (meristem, vascular bundles with fibre caps,) and the arrangement, course and linkage of the individual vascular bundles within the branch-stem-attachment. Furthermore, both ITK-SNAP and 3D-Slicer are capable of generating stl formats of the segmentation results (ITK-SNAP) or the created models (3D-Slicer) for 3D printing or subsequent finite element analysis.

#### Quantifying deformations of vascular bundles and their fibre caps

The deformations of vascular bundles and their fibre caps were quantified manually and by using digital image correlation software. The central aim was to detect differences concerning the degree and direction of displacements within certain regions of the plant ramification. The MR-images allow studying the branch-stem-attachments in three distinct orientations: axial, sagittal and coronal. Each of these image planes were subdivided into distinct regions (see Supplementary Fig. S4). The axial plane was subdivided into the axial regions left (L: width = 4 mm), centre (C: width = 4 mm) and right (R: width = 4 mm), whereas the sagittal plane was subdivided into the sagittal regions top (I: length = 5 mm), centre (II: length = 5 mm) and bottom (III: length = 5 mm). The axial plane shows low resolution areas (1 mm of thickness) close to the lateral meristem (see Supplementary Fig. S4). These low resolution areas were excluded from the distinct regions, as vascular bundles are difficult to differentiate. The coronal plane was used to determine the coronal regions close to the main stem (a) and further away from the main stem (b) (see Supplementary Fig. S4). The width of the coronal regions depended on the individual tested: DM09: width = 4.5 mm; DM10: width = 6 mm. Consequently, axial and sagittal regions form a 3×3 grid with a grid-depth of the corresponding coronal region a or b.

Manual deformation analysis: The manual deformation analysis was performed with the help of the software 3D-Slicer. A Cartesian coordinate system was determined as a reference for each imaged individual (DM09 and DM10) in order to identify the location of a given point on a vascular bundle with fibre cap in the three-dimensional space. The z-axis is in line with and placed in the centre of the main stem. The y-axis is directed towards the branch running through the centre of both axial and sagittal plane of the branch-stem-attachment region. The x-axis runs parallel to the coronal plane (see Supplementary Fig. S4).

Vascular bundles with characteristic and retrievable features (branching, fusion, turns, bumps, indentations etc.) were determined for each square of the axial-sagittal-grid and for each coronal region. In a first step vascular bundles with characteristic features were identified for the unloaded condition and each feature was labelled by using the paintbrush tool of the editor module of 3D-Slicer. In a second step the same features were labelled for the loaded condition of the plant ramification. Finally, the position of each labelled feature was measured with reference to the Cartesian coordinate system for both mechanical conditions using the ruler tool of the annotation module in 3D-Slicer. Each feature equals a point on a vascular bundle (P_1_) which is being displaced (P_2_) due to an outer applied force. The displacement of P_1_ to the position P_2_ is given by the coordinates of their vector:


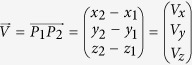


The vector coordinates Vx, Vy and Vz give the magnitude and direction of displacement along the respective coordinate axis.

Digital image correlation: For each axial region, left (L), centre (C) and right (R) (Supplementary Fig. S4), identical sagittal images of both mechanical conditions of the ramifications of both individuals of *D. marginata* were extracted from 3D-Slicer and subsequently cropped using the open access software Fiji^28^. The cropped images were then used to analyse the deformation of vascular bundles and their fibre caps using digital image correlation software (ARAMIS Professional V8 SR1) and the Fiji plugin bUnwarpJ^29^ for 2D image registration.

The aim of performing an elastic and consistent image registration using bUnwarp was to determine whether a simple 2D image registration can map deformations sufficiently. Thus, the gained deformation field image was cropped to only visualise the vector field, which matches the geometry of the plant ramification, leaving out the image registration information of the image areas surrounding the plant structure (Supplementary Fig. S6).

ARAMIS Professional V8 SR1 software was used to perform a digital image correlation on the extracted sagittal images. For each set of sagittal images (unloaded and loaded) of each axial region (L, C and R) the scale bar had to be calibrated to secure a sufficiently accurate calculation of deformations. The point based deformation analysis of the inspection function was used to select characteristic features located on the vascular bundles and their fibre caps within the unloaded sagittal image. The facet size was set to 10 or 15 pixels. The software then calculates the displacement of the selected features in x-, y- and z-direction. The displacements can be visualised as arrows which point towards the direction of displacement and their length and colour give the magnitude of displacement. An additional scale bar gives the exact values of the displacement with a colour coding. Alternatively, the free digital image correlation (DIC) GOM Correlate can be used.

Statistical analysis: Differences between the coronal regions a and b were assessed using generalized linear models with Gaussian error distribution. Vy and Vz were fitted respectively as dependent variables and whether the measurements were taken close to the main stem (coronal region a) or further away from the main stem (coronal region b) were included as fixed predictors. Additionally, it was tested whether measurements taken at the axial regions left (L), centre (C) and right (R) as well as taken at sagittal regions top (I), centre (II) or bottom (III) of the branch differ between the coronal regions a and b within each individual. Hereto the measurements Vy and Vz were again fitted as dependent variables and the location of measurement (L, C, R (Group 1) or I, II, III (Group 2)) was included as fixed predictors. Finally it was tested whether both individuals differ in their Vz values by fitting Vz as dependent variable and individual ID as fixed predictor. All analyses were performed in R version 3.0.2^39^.

## Additional Information

**How to cite this article**: Hesse, L. *et al*. Magnetic resonance imaging reveals functional anatomy and biomechanics of a living dragon tree. *Sci. Rep.* **6**, 32685; doi: 10.1038/srep32685 (2016).

## Supplementary Material

Supplementary Information
