# Supplementary material for: Magnetic resonance imaging reveals functional anatomy and biomechanics of a living dragon tree
Source: Sci Rep. 2016 Sep 8;6:32685. doi: 10.1038/srep32685 (PMC5015020; doi:10.1038/srep32685)
Supplement: Supplementary Information [file srep32685-s1.pdf]

Supplementary Information

Magnetic resonance imaging reveals functional anatomy and biomechanics of a living dragon tree

Linnea Hesse[1,2,*], Tom Masselter[1,2,3], Jochen Leupold[4], Nils Spengler[5], Thomas Speck[1,2,3], Jan Gerrit Korvink[5]

[1]Plant Biomechanics Group and Botanic Garden, University of Freiburg, Germany

[2]Freiburg Centre for Interactive Materials and Bioinspired Technologies (FIT), Germany

[3]Competence Network Biomimetics, Germany

[4]Medical Physics, Department of Radiology, University Medical Center Freiburg, Germany

[5]Institute of Microstructure Technology, Karlsruhe Institute of Technology (KIT), Germany

*Corresponding author: linnea.hesse@biologie.uni-freiburg.de

Supplementary Figures

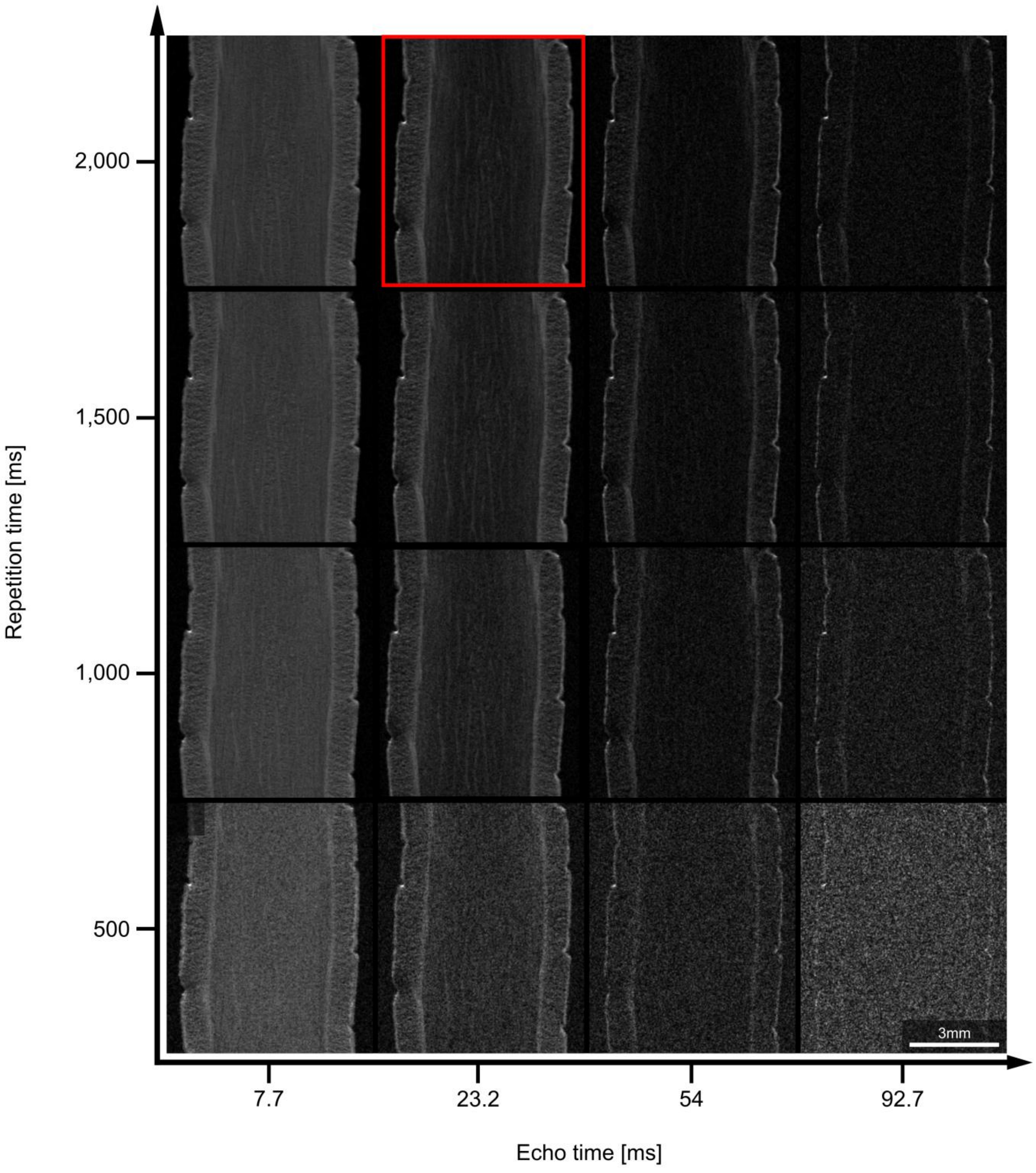

Figure S1: Spin echo (MSME) images with varying repetition time (TR) and varying echo time (TE). Altering TE and TR weights the contrast to one or several intrinsic parameters such as the water content (proton density ρ) or the degree of water binding (spin-lattice

relaxation time T1 and the spin-spin relaxation time T2). The vascular system is visualised best using T2-weighted images (TR = 2000 ms, TE = 23.2 ms; red box). Images that are weighted towards the proton density ρ (TR = 2000 ms, TE = 7.7 ms) or the spin-lattice relaxation time T1 (TR = 500 ms, TE = 7.7 ms) show only little tissue contrast. T1-weighted images (TR = 500 ms, TE = 7.7 ms) show almost no contrast between the vascular bundles with fibre caps and its adjacent tissues. Field of view: 1 cm x 1 cm; acquisition matrix: 256 x 256; slice thickness: 200 µm (for details see Table S2).

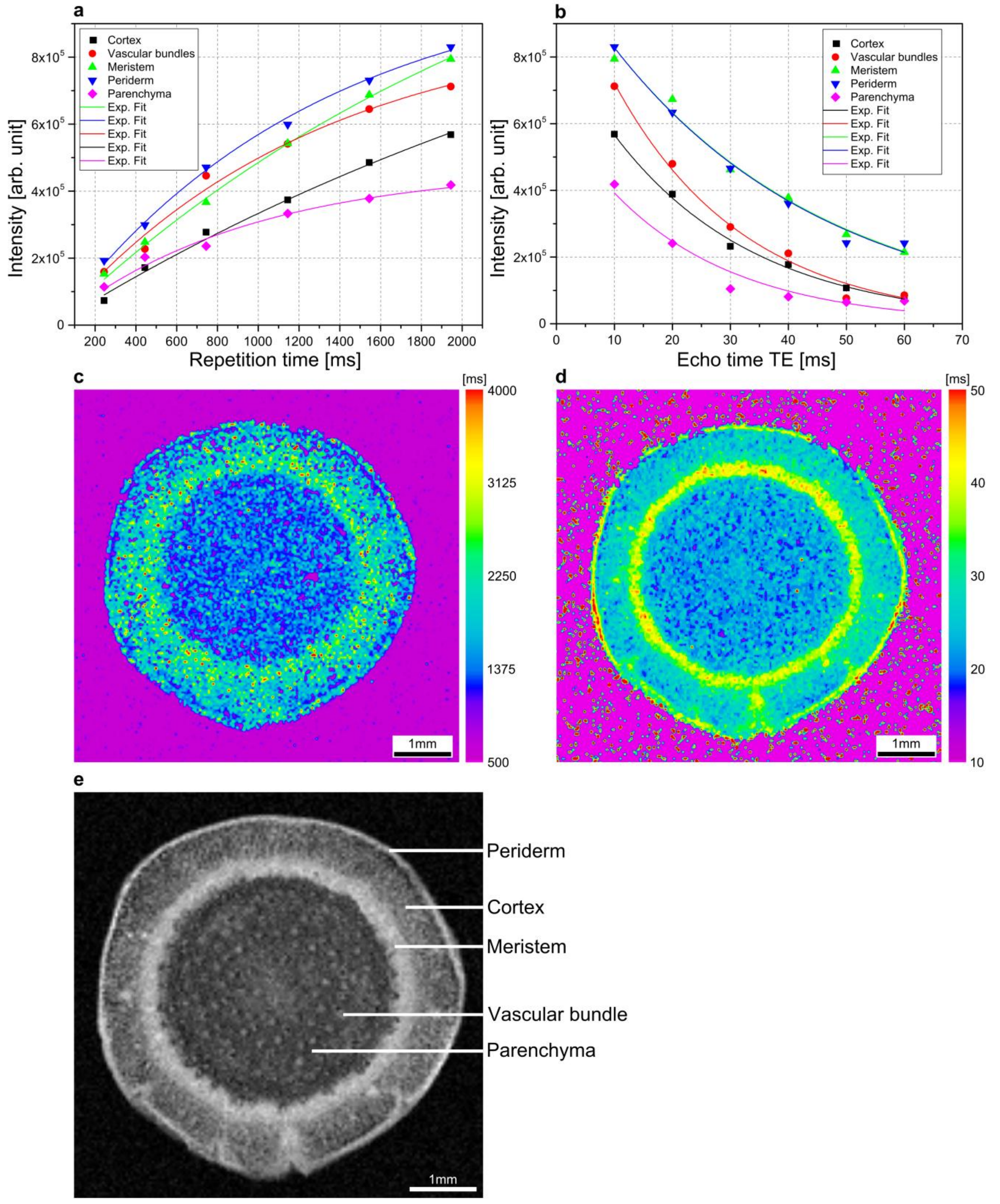

Figure S2: Tissue dependent relaxation processes. a: Representative measurement and resulting fit for each tissue. The signal intensity increases with the repetition time (TR) during T1 recovery. The signal intensity of the parenchyma and cortex are lower when compared to those of the periderm, meristem and vascular bundles with fibre caps which are quite similar

and only differ clearly with long TR. b: Representative measurement and resulting fit for each tissue. The signal intensity decreases with increasing echo time (TE) during T2 relaxation. The signal intensities of the meristematic tissue and the vascular bundles differ most when a TE of 39 ms is applied. Best contrast images of the vascular system are acquired with long TR of 2000 ms and long TE of 39 ms (compare Supplementary Fig. S1). Additionally, the T1 (c) and T2 Maps (d) are given. Respective tissues are labelled in e.

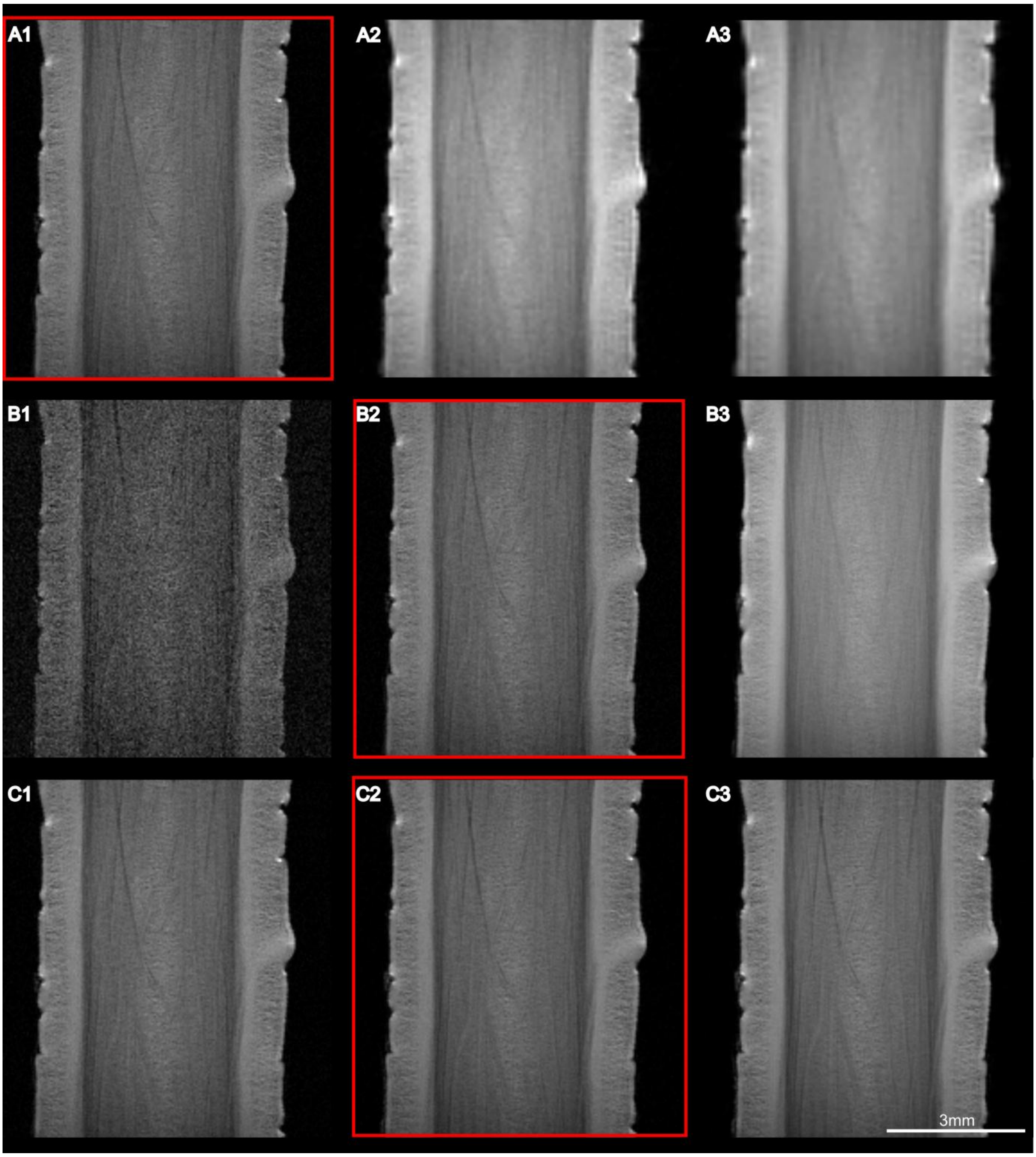

Figure S3: Influence of varying in-plane resolution (A1-A3), slice thickness (B1-B3) and number of signal excitation (C1-C3). In-plane resolution: The image A1 has the highest resolution (FOV = 1 cm^2; acquisition matrix = 256 x 256). Lowering the matrix size to 128 x 128 reduces the resolution (A2). The combination of a slightly larger FOV (1.28 cm x 1.28 cm) and a small matrix (128 x 128) reduces the resolution even further (A3). Slice thickness: A small slice thickness reduces the signal-to-noise ratio which leads to a reduction of the image resolution (B1; slice thickness = 50 µm). Choosing a slice thickness which is too large will lead to strong partial volume effects and thus to a blurring of the image (B3; slice thickness = 600 µm). A slice thickness of between 100 µm and 200 µm leads to the best imaging results (B2; slice thickness = 200 µm). For B1-B3 FOV = 1 cm2 and matrix = 256 x 256. A gradual increase of the number of signal excitations from NEX = 4 (C1) to NEX = 8 (C2) to NEX = 16 (C3) increases the signal-to-noise ratio but also the image acquisition time from 42 min to 103 min to 204 min. For C1-C3 FOV = 1 cm2 and matrix = 256 x 256. FOV: field of view; NEX: Number of signal excitation. The red boxes highlight optimal images with most suitable parameters. Details concerning the imaging parameters are listed in Supplementary Table S2.

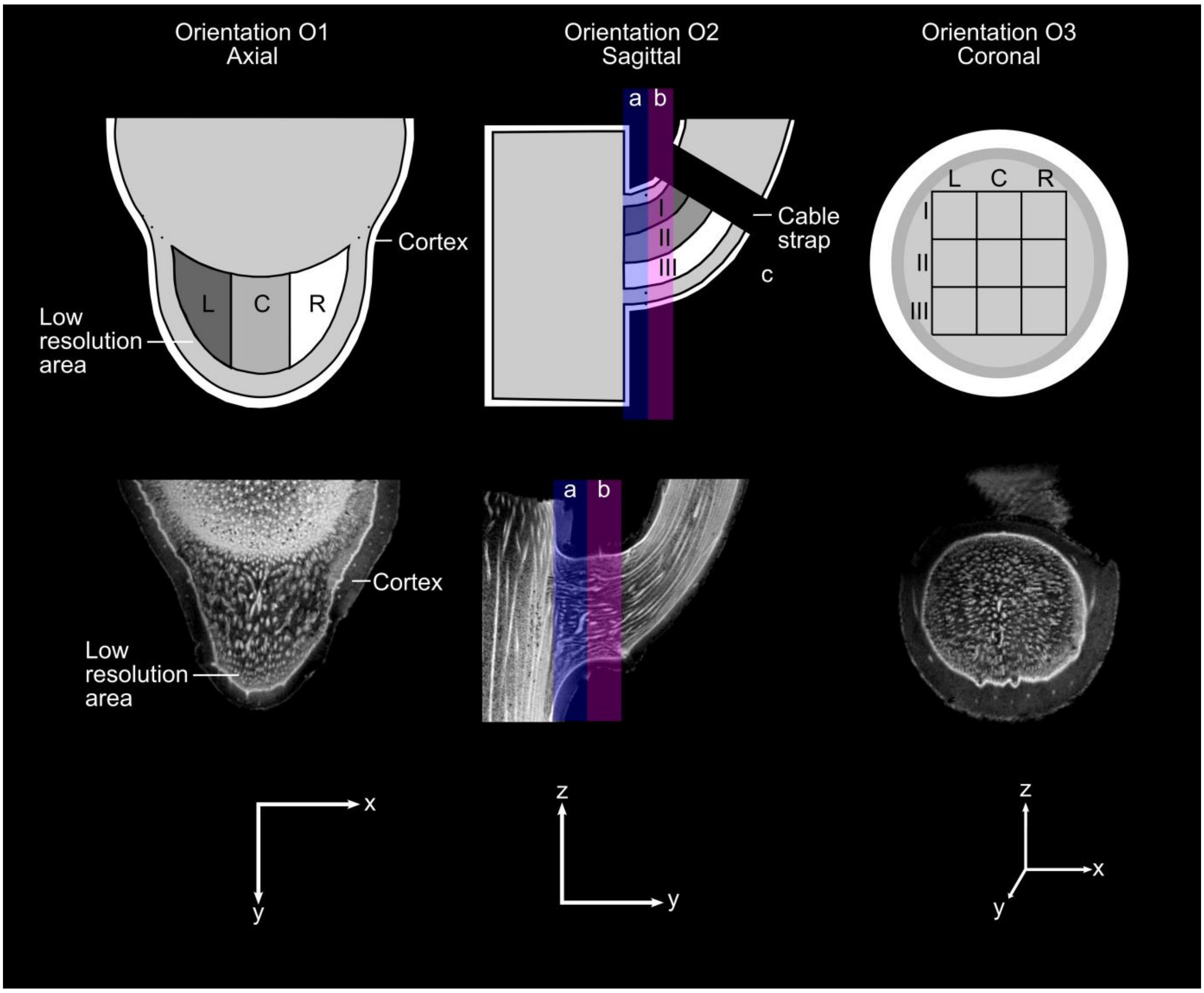

Figure S4: Schematic classification of the axial- sagittal and coronal regions. The axial plane is subdivided into the left region (L), central region (C) and the region located on the right side of the branch (R). The sagittal plane is subdivided into the upper (I), central (II) and the bottom (III) region of the branch. The coronal region is dependent on the location close to the main stem (a) and further away from the main stem (b). The x-, y- and z-axis are given for each slice orientation.

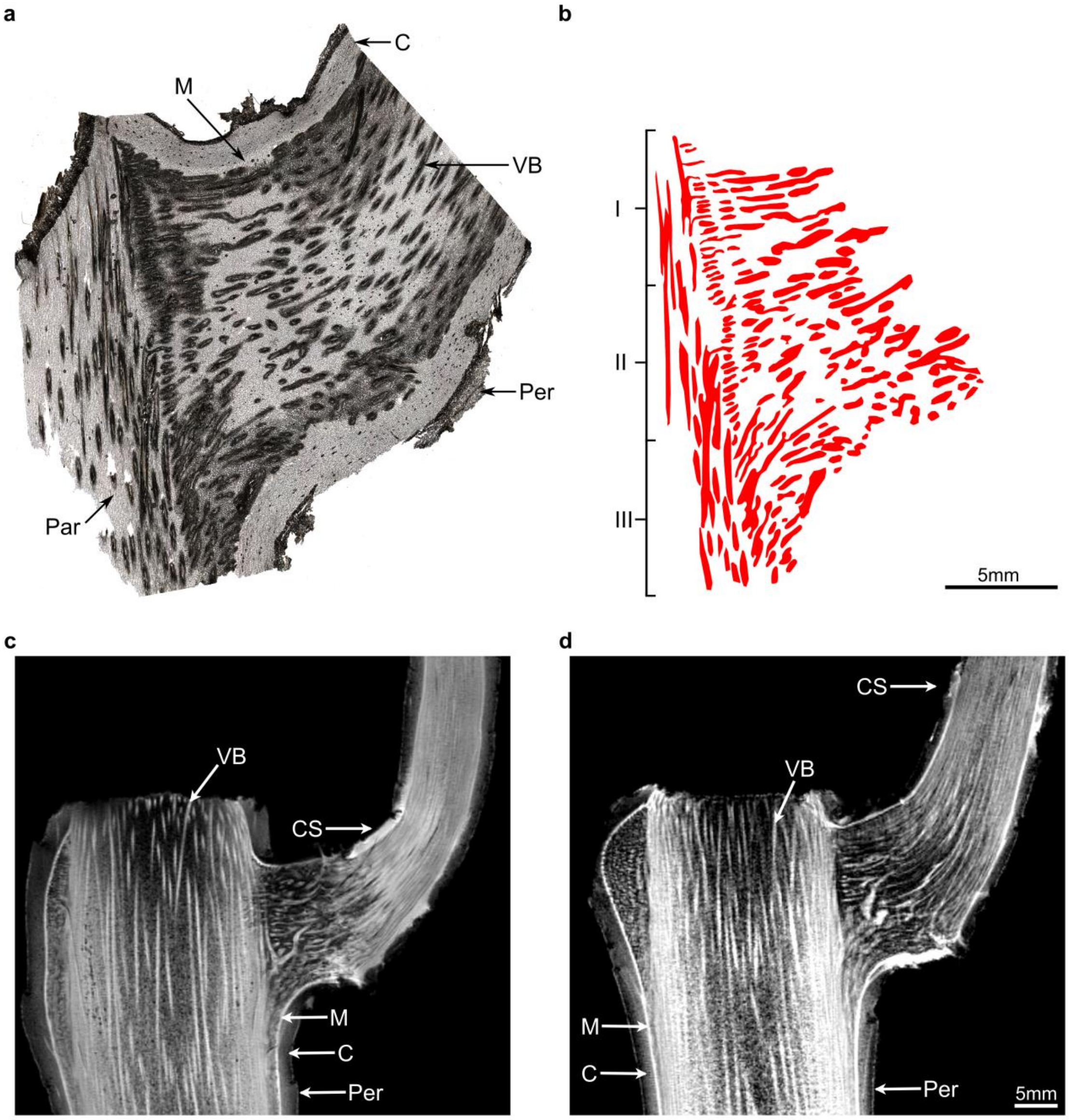

Figure S5: Overview of the anatomy of a branch stem attachment of *Dracaena marginata* and location of mechanical loading along the branches of individual plants DM09 and DM10. a: Light microscopic image of an anatomic section (sagittal) through the branch-stem-attachment of *D. marginata*. b: Segmentation image of image a showing the arrangement and orientation of vascular bundles and their fibre caps. The sagittal regions top (I), centre (II) and bottom (III) are given. c: Location of mechanical loading along the branch of individual DM09 (CS: cable strap). d: Location of mechanical loading along the branch of individual DM10 (CS: cable strap). Co: cortex; CS: cable strap; M: lateral meristem; Par; parenchyma tissue; Per: periderm; VB: vascular bundle and their fibre caps.

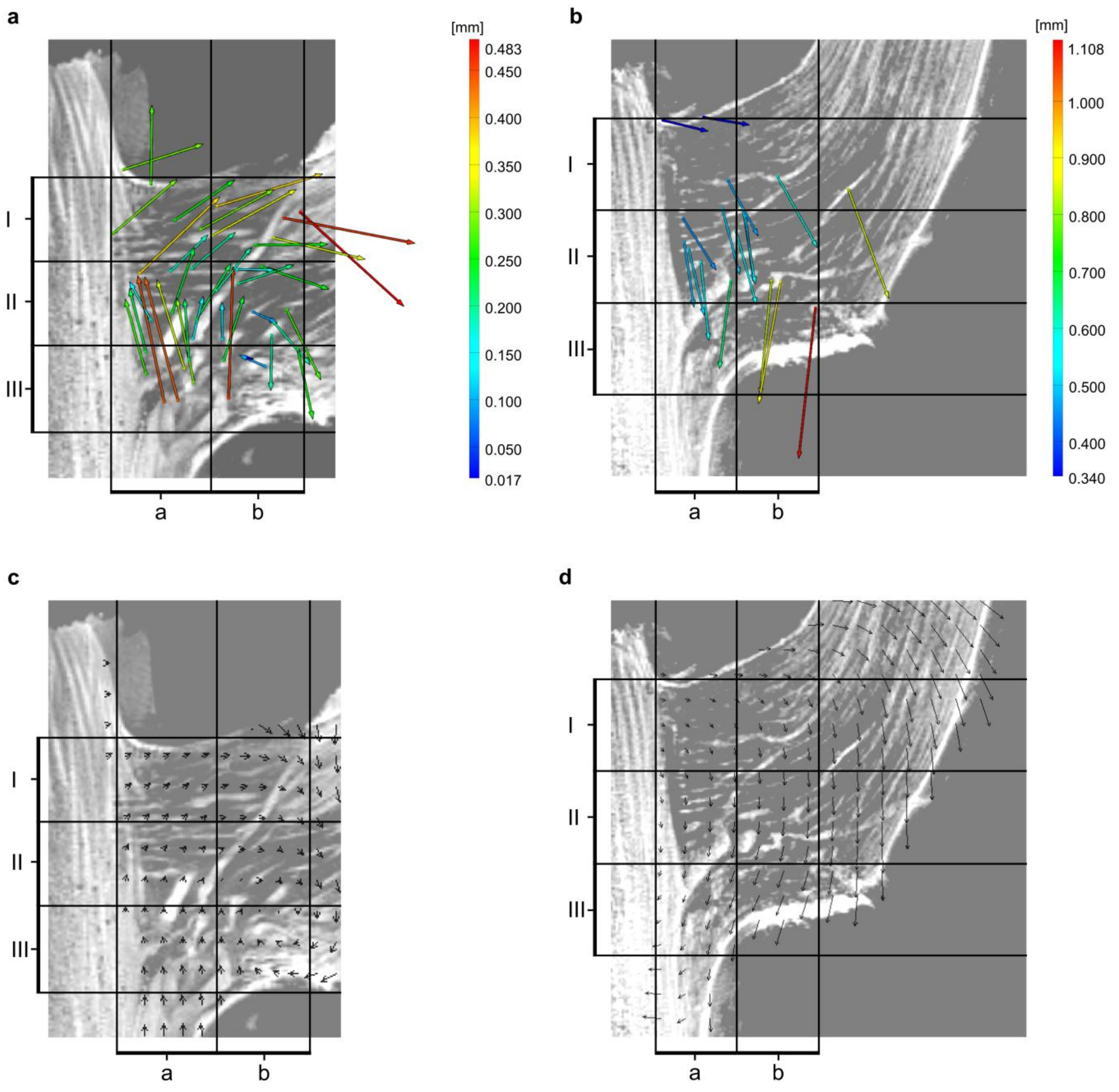

Figure S6: Visualisation of the displacement in y- (tensile and compressive strains; Vy values) and z-direction (shear and bending strains; Vz values) simultaneously. Displacement vectors in the sagittal image of the axial region C (branch centre) of individual DM09 (a) and DM10 (b) created by using the digital image correlation software ARAMIS Professional V8 SR1. c: 2D elastic and consistent image registration using the Fiji plugin bUnwarp for sagittal image of the axial region C (centre of the branch) of individual DM09 (c) and DM10 (d).

Supplementary Tables

Supplementary Table S1: Pairwise comparisons of generalized linear models analysing the effects of mechanical loadings on vascular bundles and their fibre caps located within distinct regions (axial, sagittal and coronal regions) of the branch-stem-attachment of the *Dracaena marginata* individuals DM09 and DM10. In the table it is being differentiated between a displacement along the y-axis (tensile and compressive strains; Vy values) and z-axis (shear and bending strains; Vz Values). Vascular bundles located within the axial regions left (L), centre (C) and right (R) or sagittal regions top (I), centre (II) and bottom (III) are combined with their location within the coronal region close to the main stem (a) and further away from the main stem (b). The *t*-values are given using the top row as a reference for the left column. The ANOVA for the Vy values of the axial regions did not show significance for DM10 ($X^2(5) = 1.94$, $p = 0.858$), which is why the *t*- and *P*-values are not being given.

DM09
Vy Axial Regions
$X^2(5) = 22.36$, $p < 0.001$

	**aC**		**bC**		**aL**		**bL**		**aR**	
	t	**P**	t	**P**	t	**P**	t	**P**	t	**P**
**bC**	2.68	**0.011**	-	**-**	-	**-**	-	**-**	-	**-**
**aL**	1.47	**0.150**	-0.92	**0.361**	-	**-**	-	**-**	-	**-**
**bL**	4.34	**<0.001**	1.74	**0.090**	2.47	**0.018**	-	**-**	-	**-**
**aR**	0.65	**0.518**	-1.74	**0.090**	-0.75	**0.461**	-3.26	**0.002**	-	**-**
**bR**	1.37	**0.180**	-1.14	**0.262**	-0.16	**0.878**	-2.74	**0.009**	0.62	**0.540**

DM09
Vy Sagittal Regions
$X^2(5) = 32.53$, $p < 0.001$

	**aI**		**bI**		**aII**		**bII**		**aIII**	
	t	**P**	t	**P**	t	**P**	t	**P**	t	**P**
**bI**	1.70	**0.097**	-	**-**	-	**-**	-	**-**	-	**-**
**aII**	-1.59	**0.121**	-3.68	**0.001**	-	**-**	-	**-**	-	**-**
**bII**	0.53	**0.600**	-1.31	**0.197**	2.36	**0.023**	-	**-**	-	**-**
**aIII**	-3.06	**0.004**	-5.05	**<0.001**	-1.76	**0.090**	-3.88	**<0.001**	-	**-**
**bIII**	1.13	**0.267**	-2.94	**0.006**	0.35	**0.727**	-1.76	**0.086**	-1.93	**0.061**

DM09
Vz Axial Regions

$X^2(5) = 27.13$, $p < 0.001$

	aC		bC		aL		bL		aR	
	t	P	t	P	t	P	t	P	t	P
**bC**	-3.86	**<0.001**	-	-	-	-	-	-	-	-
**aL**	0.04	**0.966**	3.50	**0.001**	-	-	-	-	-	-
**bL**	-0.94	**0.355**	2.81	**0.008**	-0.89	**0.381**	-	-	-	-
**aR**	0.56	**0.578**	4.01	**<0.001**	0.47	**0.639**	1.39	**0.172**	-	-
**bR**	-2.53	**0.016**	1.08	**0.285**	-2.33	**0.025**	-1.58	**0.121**	-2.82	**0.008**

DM09
Vz Sagittal Regions

$X^2(5) = 24.91$, $p < 0.001$

	aI		bI		aII		bII		aIII	
	t	P	t	P	t	P	t	P	t	P
**bI**	-1.78	**0.083**	-	--	-	-	-	-	-	-
**aII**	0.68	**0.501**	2.75	**0.009**	-	-	-	-	-	-
**bII**	-1.36	**0.182**	0.47	**0.638**	-2.28	**0.028**	-	-	-	-
**aIII**	2.32	**0.025**	4.33	**<0.001**	1.87	**0.069**	3.94	**<0.001**	-	-
**bIII**	-0.81	**0.421**	0.89	**0.378**	-1.57	**0.125**	0.47	**0.643**	-3.14	**0.003**

DM10
Vy Sagittal Regions

$X^2(5) = 19.66$, $p = 0.002$

	aI		bI		aII		bII		aIII	
	t	P	t	P	t	P	t	P	t	P
**bI**	-0.20	**0.841**	-	-	-	-	-	-	-	-
**aII**	-2.57	**0.012**	-2.17	**0.033**	-	-	-	-	-	-
**bII**	-1.24	**0.219**	-0.95	**0.347**	1.37	**0.176**	-	-	-	-
**aIII**	-2.62	**0.011**	-2.32	**0.023**	-0.61	**0.545**	-1.66	**0.101**	-	-
**bIII**	-3.48	**0.001**	-3.08	**0.003**	-1.26	**0.211**	-2.44	**0.017**	-0.46	**0.650**

DM10
Vz Axial Regions

$X^2(5) = 1.94$, $p = 0.858$

	aC		bC		aL		bL		aR	
	t	P	t	P	t	P	t	P	t	P
**bC**	-5.59	**<0.001**	-	-	-	-	-	-	-	-
**aL**	-1.32	**0.193**	3.74	**<0.001**	-	-	-	-	-	-
**bL**	-1.64	**0.105**	3.29	**0.002**	-0.33	**0.742**	-	-	-	-
**aR**	0.89	**0.377**	6.09	**<0.001**	2.04	**0.045**	2.33	**0.022**	-	-
**bR**	-3.98	**<0.001**	1.73	**0.089**	-2.24	**0.028**	-1.82	**0.074**	-4.59	**<0.001**

	DM10 Vz Sagittal Regions									
			$X^2(5) = 117.54$, $p < 0.001$							
	**aI**		**bI**		**aII**		**bII**		**aIII**	
	t	**P**	t	**P**	t	**P**	t	**P**	t	**P**
**bI**	-3.39	**0.001**	-	**-**	-	**-**	-	**-**	-	**-**
**aII**	-3.91	**<0.001**	-0.03	**0.979**	-	**-**	-	**-**	-	**-**
**bII**	-8.39	**<0.001**	-4.29	**<0.001**	-4.94	**<0.001**	-	**-**	-	**-**
**aIII**	-4.87	**<0.001**	-1.70	**0.093**	-1.85	**0.068**	2.03	**0.046**	-	**-**
**bIII**	-9.27	**<0.001**	-5.46	**<0.001**	-6.15	**<0.001**	-1.64	**0.105**	-3.26	**0.002**

Supplementary Table S2: Image acquisition protocols used for contrast adjustments and adjusting the in-plane resolution, the ideal slice thickness and the ideal number of excitation (NEX). A Multi slice multi echo (MSME) sequence was used to create an image series of the same slice with varying echo times (TE). The experiment (E) was repeated (No. of E) with varying the repetition times (TR = 500 – 3000 ms), the field of view (FOV: 1 cm x 1 cm or 1.28 cm x 1.28 cm) and/or matrix (256 x 256 or 128 x 128), the slice thickness (50 µm – 600 µm) or with varying NEX (4 – 16). NEX: Number of excitations; FOV: Field of view, E: Type of experimental image acquisition and parameter variation; No. of E: Number of experiment.

**E**	**No. of E**	**TR [ms]**	**TE [ms]**	**Echo train length [/]**	**FOV [cm^2]**	**No. of pixels in acquisition matrix**	**NEX**	**Slice thickness [µm]**	**No. of slices**
Contrast	1	500	7.7-92.7	12	1	256	4	200	1
	2	1000	7.7-92.7	12	1	256	4	200	1
	3	1500	7.7-92.7	12	1	256	4	200	1
	4	2000	7.7-92.7	12	1	256	4	200	1
	5	2500	4.5-53.8	12	1	256	4	200	1
	6	3000	4.5-53.8	12	1	256	4	200	1

In-plane resolution	1	3000	4.5-53.8	12	1	256	4	200	1
	2	3000	2.9-35.2	12	1.28	128	4	200	1
	3	3000	2.9-35.2	12	1	128	4	200	1
Slice thickness	1	3000	4.5-53.8	12	1	256	4	50	1
	2	3000	4.5-53.8	12	1	256	4	100	1
	3	3000	4.5-53.8	12	1	256	4	200	1
	4	3000	4.5-53.8	12	1	256	4	300	1
	5	3000	4.5-53.8	12	1	256	4	400	1
	6	3000	4.5-53.8	12	1	256	4	500	1
	7	3000	4.5-53.8	12	1	256	4	600	1
NEX	1	3000	4.5-53.8	12	1	256	4	200	1
	2	3000	4.5-53.8	12	1	256	8	200	1
	3	3000	4.5-53.8	12	1	256	16	200	1

Supplementary Methods

This supplementary method section gives a detailed step-by-step approach to generate three-dimensional models from magnetic resonance images of mechanically loaded plant structures.

ITK-SNAP: image segmentation steps

Step 1: Import of raw-data (DICOM format: .dcm).

Step 2: Saving the raw-data as main image file (NiFTI format: .nii).

Step 3: Semi-automated segmentation using the active contour segmentation (snake) mode and preprocessing the main image by adjusting the thresholding.

Step 4: Optimization of the semi-automated segmentation results using the paintbrush mode.

Step 5: Saving the resulting segmentation image in NiFTI-format (.nii).

3D-Slicer: image analysis steps

Step 1: Import of the following files into 3D Slicer:

- Main image unloaded (.nii).
- Main image loaded (.nii).
- Segmentation images unloaded:
    - vascular system (.nii).
    - meristem(.nii).
- Segmentation images loaded:
    - vascular system (.nii).
    - meristem (.nii).

Step 2: Reorientation and alignment of the image-data for the loaded condition of the ramification to the unloaded situation using the transforms module.

Step 3: Fast automated segmentation of the geometry of the ramification under unloaded and loaded condition using the threshold effect of the editor module. The threshold modulator sets the lower and upper threshold boundaries. The lower threshold boundary is altered until no background noise

is included into the label map and only the geometry of the ramification is displayed.

Step 4: Automated segmentation of the segmentation images of both unloaded and loaded conditions of the ramification. It is preceded as in step 3.

Step 5: Segmentation of particular vascular bundles of both the unloaded and loaded condition of the ramification using the paint effect of the editor module. A more ready segmentation of the same vascular bundles in both mechanical situations (unloaded and loaded) is assured in 3D-Slicer due to the possibility of an image overlay using the slice viewer controller.

Step 6: Models of all imported data (step 1) and the segmentation results of particular vascular bundles are created with the model-maker module using the label map volumes created with the editor module (step 3 to 5).

Step 7: Generating a model overlay and altering the visualization of the models and their opacity using the model module.
